# Surgical Resection Followed by Stereotactic Radiosurgery (S+SRS) Versus SRS Alone for Large Posterior Fossa Brain Metastases: A Comparative Analysis of Outcomes and Factors Guiding Treatment Modality Selection

**DOI:** 10.3390/brainsci14111059

**Published:** 2024-10-25

**Authors:** Ruth Lau, Enrique Gutierrez-Valencia, Anna Santiago, Carolyn Lai, Danyal Baber Ahmed, Parnian Habibi, Normand Laperriere, Tatiana Conrad, Barbara-Ann Millar, Mark Bernstein, Paul Kongkham, Gelareh Zadeh, David Benjamin Shultz, Aristotelis Kalyvas

**Affiliations:** 1 Department of Surgery, Division of Neurosurgery, Toronto Western Hospital, University of Toronto, Toronto, ON M5T 2S8, Canada; ruth_lau_rodriguez@hotmail.com (R.L.); carolyn.lai@medportal.ca (C.L.); danyal.ahmed23@gmail.com (D.B.A.); parnian.habibi15@gmail.com (P.H.); mark.bernstein@uhn.ca (M.B.); paul.kongkham@uhn.ca (P.K.); gelareh.zadeh@uhn.ca (G.Z.); 2 Department of Radiation Oncology, University of Toronto, Toronto, ON M5S 1A1, Canada; enrique.gutierrez@uhn.ca (E.G.-V.); norm.laperriere@uhn.ca (N.L.); tatiana.conrad@uhn.ca (T.C.); barbara-ann.millar@uhn.ca (B.-A.M.); david.shultz@uhn.ca (D.B.S.); 3 Radiation Medicine Program, Princess Margaret Cancer Centre, University Health Network, Toronto, ON M5G 2M9, Canada; 4 Department of Biostatistics, Princess Margaret Cancer Centre, University Health Network, Toronto, ON M5G 1X6, Canada; anna.santiago@uhn.ca

**Keywords:** brain metastases, posterior fossa, neurosurgery, stereotactic radiosurgery

## Abstract

Background/Objectives: Around 20% of cancer patients will develop brain metastases (BrMs), with 15–25% occurring in the posterior fossa (PF). Although the effectiveness of systemic therapies is increasing, surgery followed by stereotactic radiosurgery (S+SRS) versus definitive SRS remains the mainstay of treatment. Given the space restrictions within the PF, patients with BrMs in this location are at higher risk of brainstem compression, hydrocephalus, herniation, coma, and death. However, the criteria for treating large PF BrMs with S+SRS versus definitive SRS remains unclear. Methods: We reviewed a prospective registry database (2009 to 2020) and identified 64 patients with large PF BrMs (≥4 cc) treated with SRS or S+SRS. Clinical and radiological parameters were analyzed. The two endpoints were overall survival (OS) and local failure (LF). Results: Patients in the S+SRS group were more highly symptomatic than patients in the SRS group. Gait imbalance and intracranial pressure symptoms were 97% and 80%, and 47% and 35% for S+SRS and SRS, respectively. Radiologically, there were significant differences in the mean volume of the lesions [6.7 cm^3^ in SRS vs. 29.8 cm^3^ in the S+SRS cohort, (*p* < 0.001)]; compression of the fourth ventricle [47% in SRS vs. 96% in S+SRS cohort, (*p* < 0.001)]; and hydrocephalus [0% in SRS vs. 29% in S+SRS cohort, (*p* < 0.001)]. Patients treated with S+SRS had a higher Graded Prognostic Assessment (GPA). LF was 12 and 17 months for SRS and S+SRS, respectively. Moreover, the S+SRS group had improved OS (12 vs. 26 months, *p* = 0.001). Conclusions: A higher proportion of patients treated with S+SRS presented with hydrocephalus, fourth-ventricle compression, and larger lesion volumes. SRS-alone patients had a lower KPS, a lower GPA, and more brain metastases. S+SRS correlated with improved OS, suggesting that it should be seriously considered for patients with large PF-BrM.

## 1. Introduction

Roughly one-fifth of patients diagnosed with cancer develop brain metastases (BrMs) over the course of their disease [1,2,3,4,5,6,7]. Among these, the posterior fossa (PF) is involved in approximately 15–25% [8,9,10].

Despite the increased number of available systemic agents with central nervous system (CNS) activity [4,5,6,7,8,9,10,11,12], treatment for BrMs primarily revolves around localized approaches such as surgery and radiotherapy [5,12,13,14].

Contemporary treatment modalities for patients presenting with a limited number of BrMs chiefly comprise surgery combined with cavity stereotactic radiosurgery (S+SRS) [2,15,16,17] or definitive stereotactic radiosurgery (SRS) [17,18,19,20,21,22]. Surgery facilitates tissue diagnosis and ameliorates symptomatic mass effects [23].

The optimal treatment strategy for large posterior fossa brain metastases (PF-BrMs), defined as those with a diameter ≥ 2 cm or volume ≥ 4 cc [16,17], remains uncertain. Clinical decision making in this patient population is challenging, as the available evidence regarding the comparative efficacy of S+SRS versus SRS alone is limited [24,25,26,27]. There is a paucity of data regarding optimal treatment strategies for large PF-BrMs. Given the anatomical particularities of the PF [28], up to 8% of patients with BrM in this location are at risk of developing acute obstructive hydrocephalus due to the compression of the fourth ventricle [29,30,31], which, if not managed effectively, can lead to rapid coma and death [32,33,34]. Symptoms of raised intracranial pressure, incoordination, and gait imbalance are also common in these patients [9,35,36].

This investigation aimed to identify relevant parameters that may assist clinicians in determining whether to administer S+SRS or SRS alone in the treatment of PF-BrMs and assess the comparative outcomes between these two cohorts.

## 2. Methodology

A prospective registry database (from 2009 to 2020) was reviewed, and all patients 18 years of age or older with large BrMs (≥4 cc) treated with SRS or S+SRS, were identified.

Surgery was performed at Toronto Western Hospital, and SRS was carried out at Princess Margaret Cancer Centre. This study was approved by our local Institutional Research Ethics Board at the University Health Network (UHN).

Clinical and radiological parameters were analyzed. We examined two endpoints: overall survival (OS) and local failure (LF).

### 2.1. Treatments

Prior to any treatment, all cases were discussed at our multidisciplinary BrM clinic with neurosurgery and radiation oncology team representatives. Patients were divided into two groups: those treated with S+SRS and those treated with SRS only.

Symptomatic younger patients with a good KPS, sizeable PF BrMs, and hydrocephalus were favored for S+SRS. Instead; asymptomatic elderly patients with a lower KPS, smaller PF BrMs, and a higher number of BrMs were directed toward SRS alone.

In the S+SRS group, procedures were performed at the University Health Network (UHN), with the subsequent administration of adjuvant SRS delivered 2–4 weeks following surgical intervention and administered to the surgical cavity in 1–3 fractions. In both treatment cohorts, SRS was delivered using a Gamma Knife Radiosurgery Unit (Elekta AB, Stockholm, Sweden).

Dose fractionation (1–3 fractions) was chosen per our institutional policies and at the discretion of the staff radiation oncologist. For single-fraction SRS (SF-SRS), the dose was ≤ 4 cc/21 Gray (Gy), 4–10 cc/18 Gy, >10 cc/15 Gy for both intact lesions and surgical cavities, until the fractionated SRS (F-SRS) technique was implemented in 2017, using the ICON frameless system. Since then, the dosing criteria were 4–<8 cc 27 Gy/3, 8–<22 cc 24 Gy/3, 22–<60 cc 21 Gy/3, for patients treated with F-SRS alone as the definitive treatment; 4–<10 cc 27 Gy/3, 10–< 20 cc 24 Gy/3, and >20 cc 21 Gy/3, for patients receiving F-SRS as an adjuvant treatment.

Our protocol for the treatment simulation technique was previously described [37]. All treatments were prescribed to a median isodose line of 50% (range: 40–60) with a GTV coverage >98%. Each SRS plan was reviewed and approved by two physicists, a radiation oncologist, and a neurosurgeon.

### 2.2. Surveillance

Patients in both the S+SRS and SRS-alone groups were monitored every 8–12 weeks following SRS treatment during the initial year, and thereafter every 3–4 months if they were clinically and radiologically stable [24,38]. All patients were followed until the time of death, last follow-up, or 1 March 2022, whichever occurred first.

### 2.3. Variables

The following variables were collected: age, gender, histology of the primary tumor, presence or absence of extracranial disease, primary tumor diagnosis date, and BrM diagnosis date. Neurological death was defined according to Patchell et al. [39]. Symptoms classically affecting patients with PF lesions were divided into gait imbalance, incoordination, and raised intracranial pressure (ICP) (nausea, headache, or vomiting). Symptoms were collected at three time points: before treatment, first follow-up, and second follow-up.

Patients’ radiological features were assessed via MRI prior to undergoing definitive treatment (S+SRS or SRS alone). They included the total number of BrMs, number of PF-BrMs, target lesion volume, radiological edema, the compression of the fourth ventricle, and the presence of hydrocephalus. Radiological edema was categorized into three grades as delineated in the literature [40,41,42,43,44]. Brain MRI T2-weighted sequences were analyzed by two reviewers, and patients were grouped into grade 1 for edema smaller than tumor volume, 2 for edema equal to lesion volume, and 3 for edema exceeding the lesion volume [43,44].

Quality of life metrics, such as the Karnofsky Performance Status (KPS), Eastern Cooperative Oncology Group (ECOG) performance status, and the Graded Prognostic Assessment Score (GPA) [45], were collected at the three time points.

### 2.4. Outcomes

The two endpoints, overall survival and local failure, were measured in months from the date of initial treatment. OS was calculated as survival either from the date of SRS (for the SRS-alone group) or from the date of surgery (for the S+SRS group) to the date of death or last follow-up. LF, defined as radiographic recurrence or progression at the original site of the treated lesion, was measured from the date of either SRS or surgery to the date of the event, defining death as a competing event.

### 2.5. Statistical Methods

Statistical analyses were carried out using R version 4.2.2. Data were summarized using descriptive statistics: mean/standard deviation and median/range for continuous variables and counts and percentages for categorical variables. Patient and radiological characteristics were compared between groups using independent *t*-tests for continuous variables and Chi-squared or Fisher’s exact tests for categorical variables. The mean number of brain metastases and PF-BrMs was compared between the SRS and S+SRS groups using the Wilcoxon rank-sum test.

ECOG at pre-treatment, first follow-up, and second follow-up were compared between groups through the Wilcoxon rank-sum test for cross-sectional comparisons at each data collection point. A linear mixed effects model with a random intercept for the interaction between the group and time point was performed to evaluate ECOG change through time. The reference levels in the model were SRS for the group and pre-treatment for the time point. For the analysis of symptoms including imbalance, ICP, and incoordination, cross-sectional comparisons were conducted between groups at each data collection time point using Chi-square or Fisher’s exact tests.

Follow-up time and overall survival between groups were analyzed using the Kaplan–Meier method with the log-rank test. The analysis of overall survival was assessed using univariate and multivariate Cox proportional hazards models for variables including treatment group (SRS alone vs. S+SRS), ECOG, KPS, number of BrMs, number of PF-BrMs, extracranial disease status, total GPA score, radiological edema, and presence of symptoms before treatment. Multivariable analysis was performed to compare overall survival between treatment groups while controlling for variables that had significant univariable associations or known clinical relationships with overall survival to estimate adjusted hazards ratios.

Local failure was evaluated for PF-BrMs using Cumulative Incidence Analysis with death as a competing risk. For local failure, univariable competing risk regression models (proportional sub-distribution hazards by Fine and Gray) for potential predictors were conducted with death as a competing risk event for the variables ECOG, number of BrM, and total GPA score. *p* values ≤ 0.05 were considered statistically significant.

## 3. Results

A total of 63 patients presenting with PF-BrM treated at our institution between 2009 and 2020 were included in this study. Of these, 29 received treatment with S+SRS, while 34 were treated with definitive SRS alone. Patient characteristics are presented in Table 1.

The median age at treatment and gender distribution were similar in both groups (61 years (range: 55–71), with 61 years (range: 55–71; female: 62%) in the S+SRS group vs. 61 years (range: 51.5–70.5; female: 56%) in the SRS-only group. The most common primary tumor location was lung in both groups.

Radiologically, significant differences were noted between the two groups concerning lesion volume, the compression of the fourth ventricle, and the presence of hydrocephalus. The mean volume of the target lesion was significantly greater in the S+SRS group (15.7 ± 9.5 cc) compared to the SRS group (6.7 ± 3 cc) (*p* < 0.001). Compression of the fourth ventricle was observed in 27 patients (93%) in the S+SRS group and 16 (47%) of the SRS patients (*p* < 0.001). Additionally, eight (24%) patients in the S+SRS cohort presented with hydrocephalus, compared to no patients from the SRS cohort.

Edema measurements between groups were not significantly different. In the S+SRS cohort, edema was grade 1 (edema volume < lesion volume) in 21% (n = 6), grade 2 (edema volume = lesion volume) in 48% (n = 14), and grade 3 (edema volume > lesion volume) in 31% (n = 9) of patients. Correspondingly, in the SRS group, the percentages were 21% (n = 7), 44% (n = 15), and 24% (n = 8) for grades 1, 2, and 3, respectively.

The median GPA was 2.5 (0.5–3.5) and 2.0 (1.0–3.5) for the S+SRS and SRS groups, respectively (*p* = 0.3). The median KPS before treatment was significantly different between groups [90 for the S+SRS group vs. 70 for the SRS group (*p* = 0.03)].

**Figure 1:**
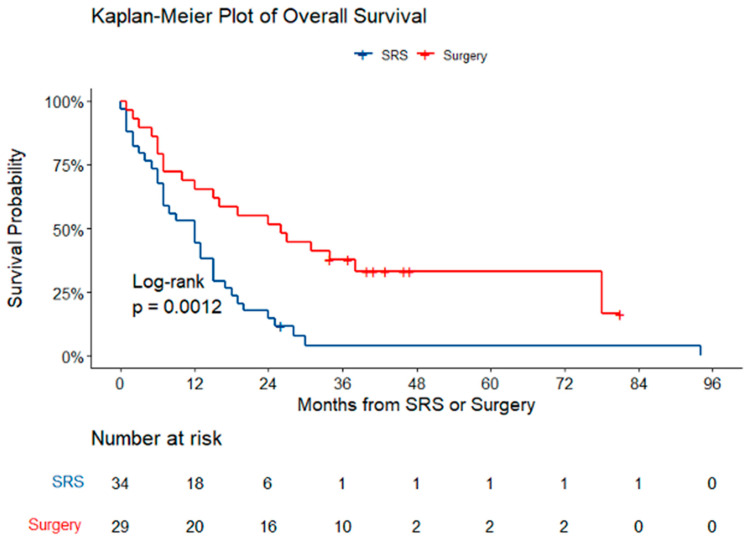
Kaplan–Meier plot of overall survival. The median survival times were 12 months for the SRS groups and 26 months for the S+SRS group.

Median follow-up was 8.5 months for the SRS group and 23.0 months for the S+SRS group. Median survival was 12 months for the SRS group and 26 months for the S+SRS group (log-rank, *p* = 0.001) (Figure 1).

On univariate analysis (UVA), treatment with S+SRS, higher KPS, and higher GPA scores were associated with better OS (*p* = 0.002, *p* = 0.003, and *p* = 0.02, respectively) (Table 2).

The multivariable model (MVA) showed a reduced hazard for S+SRS (adjusted HR = 0.40, 95% CI [0.21,0.77], *p* = 0.006) but not for the total GPA score, presence of extracranial disease, or KPS (Table 3).

The following sensitivity analyses were conducted using multivariable models for overall survival including either the GPA or KPS. The model with the KPS had a lower AIC, indicating a better model (Table 4).

While statistical significance was achieved for the adjusted hazards ratio of the KPS, the upper confidence limit includes 1.00. This confirms that neither the GPA nor KPS were more predictive than surgery regarding OS.

The rate of LF did not significantly differ between groups. Univariable competing risk regression did not identify significant predictors of LF among factors such as BrM number, extracranial disease status, total GPA score, radiological edema, and target volume (Supplementary Table S1). The 12-month cumulative incidence rates for LF, considering death as a competing risk, were 0.059 (95% CI [0.010–0.175]) for the SRS group and 0.103 (95% CI [0.103–0.247]) for the S+SRS group (*p* = 0.536). The median time to LF was 12 (4.5–17) months for SRS and 17 (7–37) months for S+SRS. One S+SRS patient required repeat SRS four months after surgery, and one patient treated with definitive SRS required salvage surgery.

**Figure 2:**
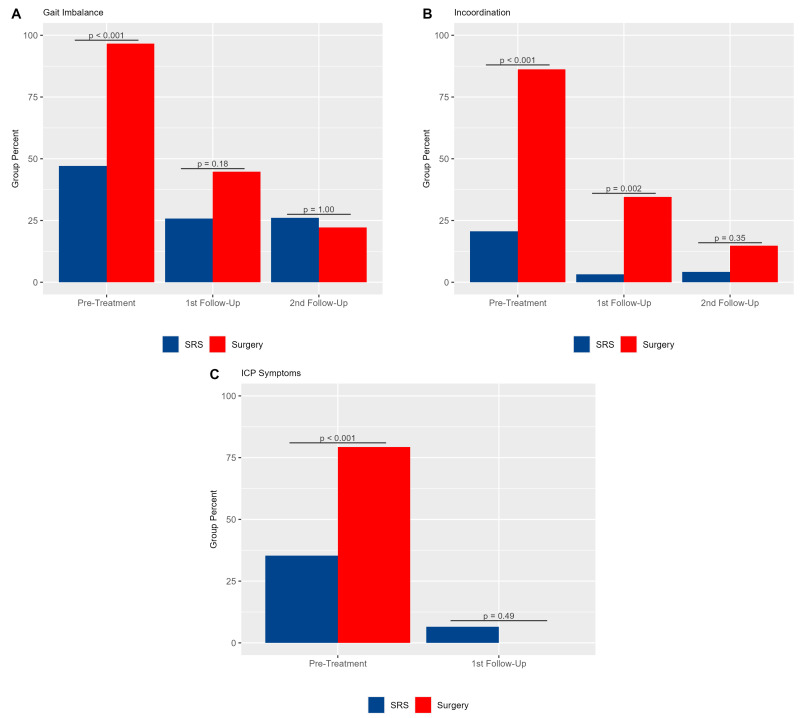
Bar chart showing the proportion of patients with (**A**) gait imbalance and (**B**) incoordination for the S+SRS and SRS groups at three time points: before treatment, at the first follow-up, and at second follow-up. (**C**) shows the proportion of patients with ICP symptoms before treatment and at first follow-up.

A higher proportion of patients presented with symptoms in the S+SRS group (29 patients, 100%) compared to the SRS group (23 patients, 68%) (*p* < 0.001). Gait imbalance was the most common symptom, reported in 28 patients (96.6%) in the S+SRS group and 16 patients (47.1%) in the SRS group (*p* < 0.001). In the S+SRS group, gait imbalance improved notably by the first follow-up, decreasing from 96.6% to 44.8% of patients, and then to 22.2% by the second follow-up. In the SRS group, gait imbalance decreased from 47.1% to 25.8% by the first follow-up and declined to 17.6% by the second follow-up (Figure 2A, Supplementary Table S2).

There was a significantly higher proportion of S+SRS patients (25 patients, 86.2%) with pre-treatment incoordination compared to SRS patients (7 patients, 20.6%) (*p* < 0.001). At first follow-up, the incoordination rate had improved in both groups from 86.2% (25 patients) to 34.5% (10 patients) in the S+SRS group and from 20.6% (7 patients) to 3.2% (1 patient) in the SRS group. Notably, at the second follow-up, there was further improvement in the S+SRS cohort, with four patients (14.8%) reporting incoordination (Figure 2B, Supplementary Table S3).

Pre-treatment ICP symptoms were more prevalent in the S+SRS cohort (*p* < 0.001). Headache, nausea, or vomiting were present in 23 patients (79.3%) and 12 patients (35.3%) in the S+SRS and SRS groups, respectively. A dramatic improvement in symptoms was observed in the first follow-up in both treatment cohorts, with no patients experiencing ICP symptoms in the S+SRS group and only two patients (6.5%) in the SRS group (Figure 2C, Supplementary Table S4).

In general, a higher proportion of patients in the S+SRS group (96.6%) experienced symptomatic improvement at the first follow-up, compared to those in the SRS group (62.5%) (Fisher’s exact test, *p* = 0.003). This trend was also observed at the second follow-up, with 92.6% of S+SRS patients showing symptomatic improvement, compared to 67.9% of SRS patients (Fisher’s exact test *p* = 0.04) (Table 5).

**Figure 3:**
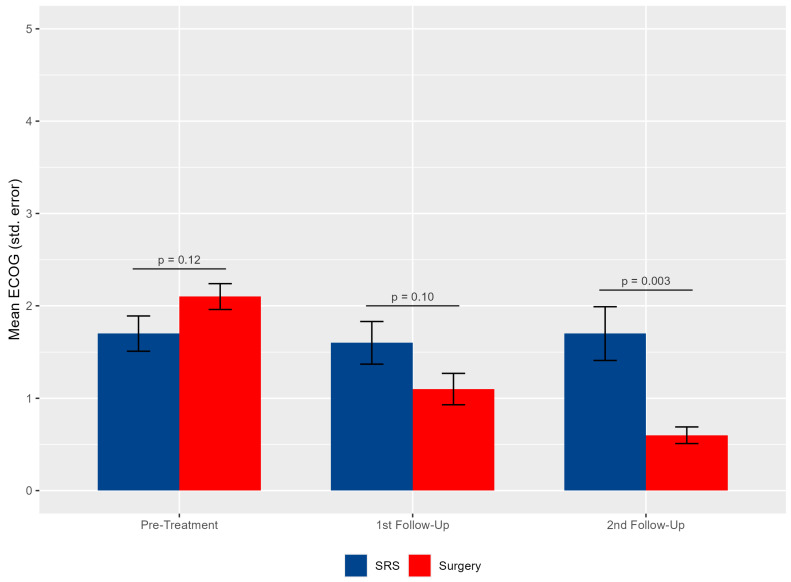
Bar graphic showing the distribution of the mean ECOG pre-treatment, at first follow-up and second follow-up.

On average, based on the Wilcoxon rank-sum test for cross-sectional analysis by time point, the SRS and SRS +S groups did not differ in ECOG scores at pre-treatment or first follow-up. The S+SRS had significantly lower ECOG scores [median: 1 (1, 1)] compared to the SRS group [median 1(1,3)] at the second follow-up (*p* = 0.003). Within-group linear mixed models show that the SRS group maintained mean ECOG scores over time. For the S+SRS group, there was a significant decrease in ECOG scores from pre-treatment to first follow-up (B = −1.00, 95% CI [−1.30, −0.70], *p* < 0.001) and from pre-treatment to second follow-up (B = −1.44, 95% CI [−1.74, −1.13], *p* < 0.001) (Figure 3, Supplementary Table S5).

## 4. Discussion

There is no consensus regarding the optimal therapeutic approach for large BrMs [24], especially when situated in the PF. Addressing this knowledge gap was the primary goal of our analysis.

In this report, we present our single-institution, retrospective experience managing patients with large PF-BrMs, contrasting patient and treatment outcomes following S+SRS versus definitive SRS. BrMs can markedly impair the quality of life for oncology patients, and PF lesions carry high risks [7,26,27,28,29,30,31,32].

Our cohort’s median LF time (12 months for SRS and 17 months for S+SRS) exceeded the six months reported in other BrM series [46]. In agreement with the existing literature, our study did not demonstrate differences in local failure for large PF-BrMs between S+SRS or SRS alone [47,48], even though larger volumes, as seen in the SRS+S group, often correlate with augmented local failure risks [49].

The mean OS for our S+SRS group was 26 months, surpassing the 15.6 months [46] and 12.2 months [48] reported in other series. The 12-month OS of our SRS cohort aligns with the established literature [50].

Our data indicate prolonged survival in the S+SRS group. Univariate OS analysis revealed associations with surgical treatment, elevated GPA scores, and higher KPSs. The presence of extracranial disease neared significance, thus warranting its inclusion in subsequent analyses (Figure 3). While the literature has linked the initial KPS, adjuvant chemotherapy or radiotherapy [33], and GPA [8] with improved survival in PF-BrM patients, direct comparisons between S+SRS and SRS treatments for large PF-BrMs are scarce. In our assessment, MVA identified only surgical intervention as an independent factor for improved OS (Table 4). The enhanced outcomes for the surgical group might be partially attributed to patient selection; surgical candidates typically possessed superior pre-treatment KPSs and fewer PF-BrMs. Furthermore, these patients experienced significant symptom alleviation during follow-up, potentially facilitating aggressive extracranial or future intracranial disease treatments and, consequently, better survival [33].

The S+SRS and SRS cohorts in our study were similar with respect to demographic characteristics, although the KPS was higher in the S+SRS cohort. This aligns with the broader literature indicating that S+SRS cohorts have superior KPSs compared to non-S+SRS cohorts. Given the inherent risks of surgical procedures, those with a higher functional status are often selected for such interventions over those in a more compromised state [51,52,53].

In contrast, our S+SRS cohort presented more pronounced symptoms compared to the SRS-only group. Notably, post-treatment symptom improvement was observed across both groups at subsequent follow-ups. However, the S+SRS cohort exhibited a more significant alleviation of symptoms. These improvements in clinical presentation correlated with lower ECOG scores. While the SRS group’s ECOG scores remained relatively stable, the S+SRS group demonstrated a marked decrease over time, culminating in a notably lower ECOG score than the SRS group by the second follow-up (Figure 2).

Overall, these findings reflect that S+SRS is typically reserved for cases where urgent symptom relief is paramount. In agreement with the previous literature [35,47], our findings underscore the efficacy of surgical resection in swiftly ameliorating such symptoms. It is important that these benefits are integrated into the patient-centric decision-making dialog when discussing treatment modalities [54].

All patients with hydrocephalus were directed to surgical management, taking into consideration the gravity of untreated obstructive hydrocephalus. Post-SRS edema can potentially worsen this condition, leading to life-threatening consequences [55]. For this reason, patients presenting with larger lesion volumes and notable compression of the fourth ventricle gravitated toward surgery.

Our cohort’s median LF time (12 months for SRS and 17 months for S+SRS) exceeded the 6 months reported in other BrM series [46]. In agreement with the existing literature, our study did not demonstrate differences in LF for large PF-BrMs between S+SRS or SRS alone [47,48], even though larger volumes, as seen in the surgical group, often correlate with augmented local failure risks [49].

The mean OS for our surgical group was 26 months, surpassing the 15.6 months [46] and 12.2 months [48] reported in other series. The 12-month OS of our SRS cohort aligns with the established literature [50].

## 5. Limitations

The modest sample size may limit our ability to draw conclusive interpretations. While the cohorts were relatively balanced, reinforcing the comparison, the findings should be approached with caution. Furthermore, the retrospective nature of our investigation, despite deriving from a prospective database, might introduce uncontrolled confounding variables. It is also notable that several patients, potentially being monitored by their local radiation oncologists, were lost to follow-up. Nonetheless, our analysis offers insights from an unfiltered sample of patients treated for PF-BrM.

## 6. Conclusions

The management of large BrMs in the critical region of the posterior fossa remains a subject of ongoing debate. Our study indicates that a higher proportion of S+SRS in patients presented with hydrocephalus, significant fourth-ventricle compression, and larger lesion volumes. In contrast, SRS-alone patients had lower KPSs, lower GPAs, and, on average, a larger number of BrMs. S+SRS correlated with enhanced overall survival on multivariable regression, suggesting that S+SRS should strongly be considered in patients with large PF tumors, perhaps even regardless of symptoms. However, given the inherent limitations of our study, these findings call for cautious interpretation and underscore the need for larger, prospective evaluations.

## Figures and Tables

**Table 1:** Patient clinical and radiological characteristics: age, sex, primary tumor site, number of brain metastases, number of posterior fossa brain metastases, KPS, ECOG, radiological edema score, target volume, compression of the fourth ventricle and hydrocephalus (BM: brain metastases; PF: posterior fossa; KPS: Karnofsky Performance Score; ECOG: Eastern Cooperative Oncology Group).

	Full Sample (n = 63)	SRS (n = 34)	Surgery (n = 29)	*p*-Value
**Age**				0.66
Mean (sd)	62.1 (12.3)	61.4 (12.8)	62.8 (11.9)	
Median (Q1, Q3)	61 (54, 71)	61.0 (51.5, 70.5)	61 (55, 71)	
Range (min, max)	(33, 88)	(34, 87)	(33, 88)	
**Sex**				0.81
Male	26 (41)	15 (44)	11 (38)	
Female	37 (59)	19 (56)	18 (62)	
**Primary Tumor Site**				0.09
Bladder	1 (2)	0 (0)	1 (3)	
Breast	10 (16)	5 (15)	5 (17)	
Cervix	1 (2)	0 (0)	1 (3)	
Colorectum	8 (13)	2 (6)	6 (21)	
Endocrine	1 (2)	1 (3)	0 (0)	
Endometrium	1 (2)	0 (0)	1 (3)	
Head and Neck	1 (2)	1 (3)	0 (0)	
Lung	22 (35)	14 (41)	8 (28)	
Melanoma	7 (11)	3 (9)	4 (14)	
Prostate	2 (3)	0 (0)	2 (7)	
Renal	5 (8)	5 (15)	0 (0)	
Sarcoma	1 (2)	1 (3)	0 (0)	
Two Primary	1 (2)	1 (3)	0 (0)	
Upper GI	2 (3)	1 (3)	1 (3)	
**Number of BMs**				0.06
Mean (sd)	2.3 (1.8)	2.5 (1.7)	2.0 (1.9)	
Median (Q1, Q3)	2 (1, 3)	2.0 (1.0, 3.8)	1 (1, 3)	
Range (min, max)	(1, 10)	(1, 8)	(1, 10)	
**Number of PF BMs**				**0.05**
Mean (sd)	1.3 (0.6)	1.4 (0.7)	1.1 (0.4)	
Median (Q1, Q3)	1 (1, 1)	1.0 (1.0, 1.8)	1 (1, 1)	
Range (min, max)	(1, 4)	(1, 4)	(1, 3)	
**KPS**				**0.03**
Mean (sd)	78.1 (18.9)	73.5 (21.3)	83.4 (14.2)	
Median (Q1, Q3)	90 (70, 90)	70 (70, 90)	90 (70, 90)	
Range (min, max)	(20, 100)	(20, 100)	(50, 100)	
**ECOG**				0.12
Mean (sd)	1.9 (1.0)	1.7 (1.1)	2.1 (0.8)	
Median (Q1, Q3)	2 (1, 3)	2 (1, 2)	2 (2, 3)	
Range (min, max)	(0, 4)	(0, 4)	(1, 3)	
**Total GPA Score**				0.3
Mean (sd)	2.2 (0.8)	2.1 (0.8)	2.3 (0.8)	
Median (Q1, Q3)	2.5 (1.5, 2.5)	2.0 (1.5, 2.5)	2.5 (1.5, 3)	
Range (min, max)	(0.5, 3.5)	(1.0, 3.5)	(0.5, 3.5)	
**Neurological Death**				0.12
0	50 (79.4)	24 (70.6)	26 (89.7)	
1	13 (20.6)	10 (29.4)	3 (10.3)	
**Radiological Edema Score**				0.50
0 or 1	17 (27)	11 (32)	6 (21)	
2 or 3	45 (73)	23 (68)	22 (79)	
Missing	1	0	1	
**Target Volume (cm^3^)**				**<0.001**
Mean (sd)	10.9 (8.1)	6.7 (3.0)	15.7 (9.5)	
Median (Q1, Q3)	8.2 (5.2, 14.5)	5.4 (4.6, 8.0)	15.2 (9.8, 19.8)	
Range (min, max)	(4.1, 54.0)	(4.1, 14.8)	(5, 54)	
**Maximum Diameter**				**<0.0001**
Mean (sd)	2.6 (0.9)	2.1 (0.5)	3.3 (0.8)	
Median (Q1, Q3)	0.9 (2.0, 3.0)	2.11 (1.8, 2.5)	2.11 (2.61, 3.67)	
Range (min, max)	(0.65, 5.21)	(0.65, 2.94)	(2.01, 5.21)	
**Fourth-Ventricle Compression**				**<0.001**
No	19 (31.1)	18 (52.9)	1 (3.7)	
Yes	42 (68.9)	16 (47.1)	26 (96.3)	
Missing	2	0	2	
**Hydrocephalus**				**0.002**
No	54 (88.5)	34 (100.0)	20 (74.1)	
Yes	7 (11.5)	0 (0.0)	7 (25.9)	
Missing	2	0	2	

**Table 2:** Univariate analysis of overall survival. Treatment surgery groups, where higher GPA scores and higher KPSs show lower hazards (better overall survival) in univariate models. (PF: posterior fossa; KPS: Karnofsky Performance Score; ECOG: Eastern Cooperative Oncology Group).

Univariable Cox Proportional Hazards Models				
	HR (95% CI)	*p*-Value	Global *p*-Value	n
**Treatment Group**		**0.002**		63
SRS	Reference			34
Surgery	0.39 (0.22, 0.71)			29
**ECOG**	1.04 (0.77, 1.41)	0.78		63
**KPS**	0.98 (0.97, 0.99)	**0.003**		63
**Number of brain metastases**	0.94 (0.80, 1.10)	0.44		63
**Number of PF brain metastases**	0.86 (0.53, 1.40)	0.55		63
**Extracranial disease status**		0.07		63
None	Reference			32
Present	1.66 (0.96, 2.89)			31
**Total GPA score**	0.65 (0.46, 0.93)	**0.02**		63
**Radiological edema grade**		0.80		62
0 or 1	Reference			17
2 or 3	0.92 (0.50, 1.71)			45
**Target volume (cm^3^)**	1.01 (0.97, 1.05)	0.78		63
**Presence of symptoms**		0.43		63
No	Reference			11
Yes	0.75 (0.38, 1.52)			52

**Table 3:** MVA overall survival: patients in the surgical group with higher GPA scores have significantly better overall survival (GPA: Graded Prognostic Assessment; KPS: Karnofsky Performance Score).

Multivariable Cox Proportional Hazards Models			
	HR (95% CI)	*p*-Value	VIF
**Group**		**0.006**	1.18
SRS	Reference		
Surgery	0.40 (0.21, 0.77)		
**Total GPA score**	0.84 (0.48, 1.47)	0.54	2.52
**extracranial disease binary**		0.22	1.81
None	Reference		
Present	1.59 (0.76, 3.36)		
**KPS**	0.99 (0.97, 1.01)	0.25	2.04

AIC = 351.7654.

**Table 4:** Sensitivity analyses using MVA models for OS that included GPA (**A**) or KPS (**B**).

**(A) Model A with Total GPA Score**			
	**HR (95% CI)**	***p*-Value**	**VIF**
**Group**		**<0.001**	1.02
SRS	Reference		
Surgery	0.36 (0.19, 0.65)		
**Extracranial disease**		0.46	1.38
None	Reference		
Present	1.28 (0.66, 2.45)		
**Total GPA score**	0.67 (0.45, 1.01)	0.057	1.38
**(B) Model B with KPS**			
	**HR (95% CI)**	***p*-Value**	**VIF**
**Group**		**0.008**	1.08
SRS	Reference		
Surgery	0.43 (0.23, 0.80)		
**Extracranial disease**		**0.032**	1.04
None	Reference		
Present	1.85 (1.05, 3.26)		
**KPS**	0.98 (0.97, 1.00)	**0.028**	1.08

(**A**) AIC = 351.0942. (**B**) AIC = 350.1379.

**Table 5:** Improvement in symptoms at first follow-up (1st FU) and second follow-up (2nd FU).

	Full Sample (n = 63)	SRS (n = 34)	Surgery (n = 29)	*p*-Value	StatTest
**Improvement in symptoms at 1st FU**				**0.003**	Fisher Exact
No	10 (18.9)	9 (37.5)	1 (3.4)		
Yes	43 (81.1)	15 (62.5)	28 (96.6)		
Missing	10	10	0		
**Improvement in symptoms at 2nd FU**				**0.04**	Fisher Exact
No	11 (20.0)	9 (32.1)	2 (7.4)		
Yes	44 (80.0)	19 (67.9)	25 (92.6)		
Missing	8	6	2		

## Data Availability

The original contributions presented in the study are included in the article/Supplementary Materials, further inquiries can be directed to the corresponding author.

## Supplementary Materials

The following supporting information can be downloaded at https://www.mdpi.com/article/10.3390/brainsci14111059/s1, Table S1: Univariate analysis of local failure for variables ECOG, number of intracranial metastases, status of extracranial disease, total GPA, radiological edema and target volume. Table S2: Gait imbalance at three time points: pre-treatment, at first follow-up and at second follow-up. Table S3: Incoordination at three time points: pre-treatment, at first follow-up and at second follow-up. Table S4: ICP symptoms at three time points: pre-treatment, at first follow-up and at second follow-up. Table S5: ECOG before treatment, at first follow-up and at second follow-up.
