# Supplementary material for: Surgical Resection Followed by Stereotactic Radiosurgery (S+SRS) Versus SRS Alone for Large Posterior Fossa Brain Metastases: A Comparative Analysis of Outcomes and Factors Guiding Treatment Modality Selection
Source: Brain Sci. 2024 Oct 25;14(11):1059. doi: 10.3390/brainsci14111059 (PMC11592184; doi:10.3390/brainsci14111059)
Supplement: Supplementary file 1 [file brainsci-14-01059-s001.zip › brainsci-3219344-supplementary.pdf]

SUPPLEMENTARY TABLES

SUPPLEMENTARY TABLE S1

	HR(95%CI)	p-value	N
**ECOG**	0.75 (0.40, 1.40)	0.36	63
**Number of Mets**	0.66 (0.34, 1.27)	0.21	63
**Extracranial Disease Status**		0.75	63
None	Reference		32
Present	0.81 (0.22, 2.94)		31
**Total GPA score**	2.12 (0.83, 5.40)	0.12	63
**Radiological Edema Grade**		0.76	62
0/1	Reference		17
2/3	1.28 (0.26, 6.28)		45
**Target Volume cm3**	1.00 (0.95, 1.06)	0.97	63

Supplementary Table S1. Univariate analysis of local failure for variables ECOG, number of intracranial metastases, status of extracranial disease, total GPA, radiological edema and target volume (ECOG: Eastern Cooperative Oncology Group; GPA: Graded Prognostic Assessment).

SUPPLEMENTARY TABLE S2

	Full Sample (n=63)	SRS (n=34)	Surgery (n=29)	p-value	StatTest
**Gait imbalance pre treatment**				**<0.001**	Fisher Exact
No	19 (30.2)	18 (52.9)	1 (3.4)		
Yes	44 (69.8)	16 (47.1)	28 (96.6)		
**Imbalance 1st FU**				0.18	Fisher Exact
No	39 (65.0)	23 (74.2)	16 (55.2)		
Yes	21 (35.0)	8 (25.8)	13 (44.8)		
Missing	3	3	0		
**Imbalance 2nd FU**				1.00	Fisher Exact
No	38 (76.0)	17 (73.9)	21 (77.8)		
Yes	12 (24.0)	6 (26.1)	6 (22.2)		
Missing	13	11	2		

Supplementary Table S2. Gait imbalance at three time points: pre-treatment, at first follow-up and at second follow-up (FU: Follow-Up).

SUPPLEMENTARY TABLE S3

	Full Sample (n=63)	SRS (n=34)	Surgery (n=29)	p-value	StatTest
**Incoordination pre treatment**				**<0.001**	Fisher Exact
No	31 (49.2)	27 (79.4)	4 (13.8)		
Yes	32 (50.8)	7 (20.6)	25 (86.2)		
**Incoordination 1st FU**				**0.002**	Fisher Exact
No	49 (81.7)	30 (96.8)	19 (65.5)		
Yes	11 (18.3)	1 (3.2)	10 (34.5)		
Missing	3	3	0		
**Incoordination 2nd FU**				0.35	Fisher Exact
No	46 (90.2)	23 (95.8)	23 (85.2)		
Yes	5 (9.8)	1 (4.2)	4 (14.8)		
Missing	12	10	2		

Supplementary Table S3. Incoordination at three time points: pre-treatment, at first follow-up and at second follow-up. (FU: Follow-Up)

SUPPLEMENTARY TABLE S4

	Full Sample (n=63)	SRS (n=34)	Surgery (n=29)	p-value	StatTest
**ICP symptoms pre treatment**				**<0.001**	Fisher Exact
No	28 (44.4)	22 (64.7)	6 (20.7)		
Yes	35 (55.6)	12 (35.3)	23 (79.3)		
**ICP symptoms 1st FU**				0.49	Fisher Exact
No	58 (96.7)	29 (93.5)	29 (100.0)		
Yes	2 (3.3)	2 (6.5)	0 (0.0)		
Missing	3	3	0		
**ICP symptoms 2nd FU**				1.00	Fisher Exact
No	50 (100.0)	23 (100.0)	27 (100.0)		
Yes	0 (0.0)	0 (0.0)	0 (0.0)		
Missing	13	11	2		

Supplementary Table S4. ICP symptoms at three time points: pre-treatment, at first follow-up and at second follow-up. (ICP: Intracranial Pressure, FU: Follow-Up)

SUPPLEMENTARY TABLE S5

	Full Sample (n=63)	SRS (n=34)	Surgery (n=29)	p-value	StatTest
**ecog**				0.12	Wilcoxon Rank Sum
Mean (sd)	1.9 (1.0)	1.7 (1.1)	2.1 (0.8)		
Median (Q1,Q3)	2 (1, 3)	2 (1, 2)	2 (2, 3)		
Range (min, max)	(0, 4)	(0, 4)	(1, 3)		
**ECOG 1st FU**				0.10	Wilcoxon Rank Sum
Mean (sd)	1.4 (1.1)	1.6 (1.3)	1.1 (0.9)		
Median (Q1,Q3)	1 (1, 2)	1 (1, 3)	1 (1, 1)		
Range (min, max)	(0, 4)	(0, 4)	(0, 3)		
Missing	3	3	0		
**ECOG 2nd FU**				**0.003**	Wilcoxon Rank Sum
Mean (sd)	1.1 (1.1)	1.7 (1.4)	0.6 (0.5)		
Median (Q1,Q3)	1 (0, 1)	1.0 (1.0, 2.5)	1 (0, 1)		
Range (min, max)	(0, 4)	(0, 4)	(0, 1)		
Missing	13	11	2		

Supplementary Table S5. ECOG before treatment, at first follow-up and at second follow-up (ECOG: Eastern Cooperative Oncology Group; FU: Follow-Up).
